# Brain-wide identification of LIN-41 (TRIM71) protein-expressing neurons by NeuroPAL

**DOI:** 10.17912/micropub.biology.000472

**Published:** 2021-09-23

**Authors:** Mushaine Shih, Chieh Chang

**Affiliations:** 1 Department of Biological Sciences, University of Illinois at Chicago

## Abstract

LIN-41 (TRIM71), an ancient protein best known for its role in timing mitotic stem cell lineages, has been recently shown to be involved in postmitotic neurons to time their differentiation and post-differentiation. Here, we report the identification of 276 LIN-41 protein-expressing neurons in the *C. elegans* nervous system by NeuroPAL and a CRISPR-engineered mNG::LIN-41 reporter, which represents 91% of all hermaphrodite neurons and includes 87 neurons that were not previously reported by CeNGEN using single-cell RNA-seq. Broad *lin-41* protein expression in *C. elegans* neurons suggests a widespread role of LIN-41 (TRIM71) in timing neuronal assembly, plasticity, and maintenance.

**Figure 1. Broad lin-41 expression in the C. elegans nervous system at the third larval stage:**
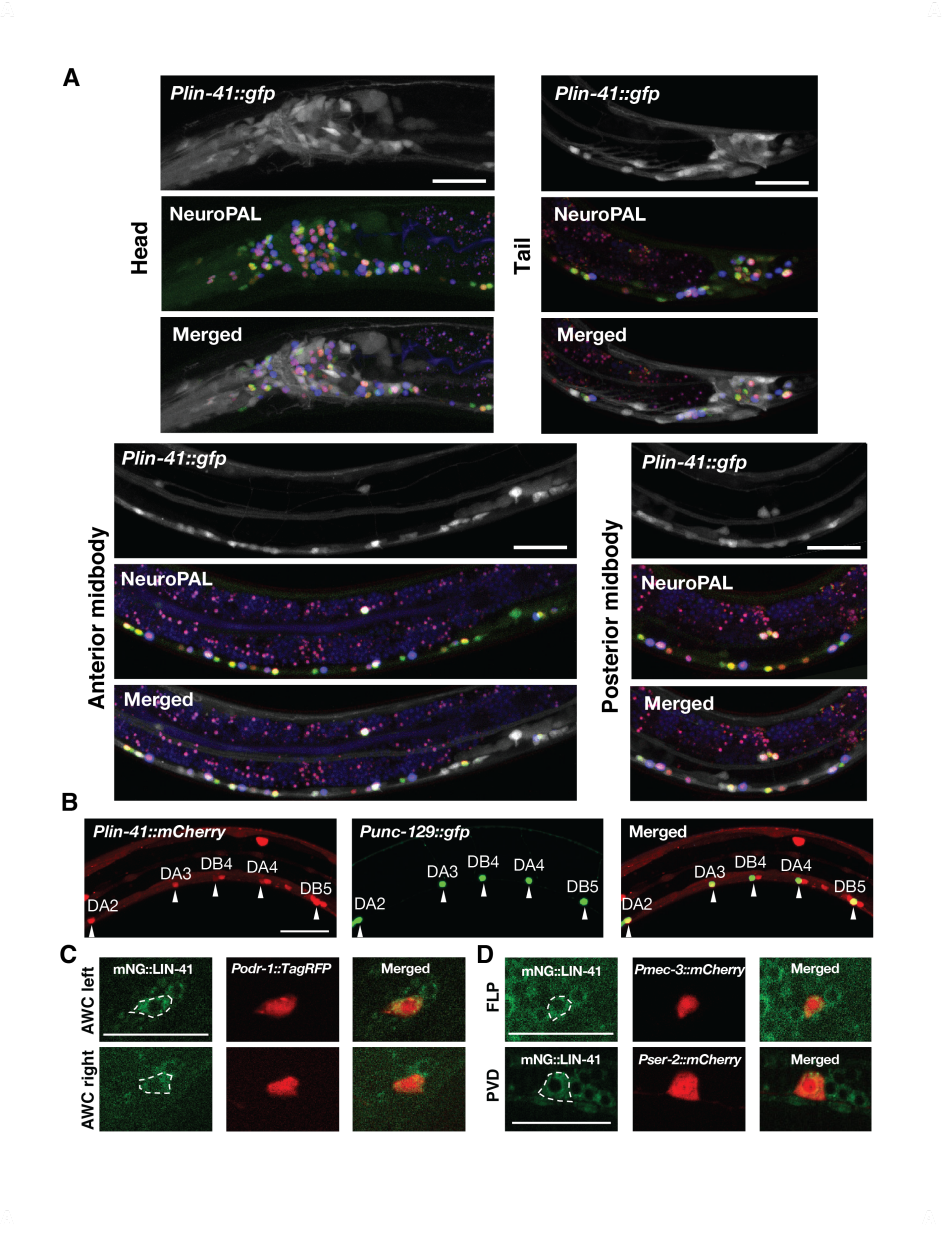
( **A** ) NeuroPAL markers were used to identify *lin-41* -expressing neurons based on a *Plin-41::gfp* reporter expression in the head, anterior midbody, posterior midbody, and tail regions. ( **B** ) *lin-41* expression in DA/B motor neurons was verified using a *Punc-129::gfp* reporter. Arrowheads point to DA/B motor neurons. A CRISPR-engineered reporter strain with the endogenous *lin-41* gene tagged ( *lin-41(xr76)* [mNG::LIN-41]) was used to identify perinuclear expression of LIN-41 proteins in a pair of AWC olfactory neurons (AWC ^left^ and AWC ^right^ ) ( **C** ), and FLP and PVD nociceptive neurons ( **D** ). Scale bars represent 20 μm.

## Description

LIN-41 (TRIM71) proteins, which are evolutionarily conserved and best known for their roles in the timing of events in mitotic stem cell lineages, have been recently shown to be reutilized in postmitotic neurons to time differentiation and post-differentiation events. The importance of LIN-41 in the nervous system of *C. elegans* has begun to emerge, which includes temporal regulation of developmental decline in neuronal regeneration, sexually dimorphic nervous system differentiation, and sexual maturation of the male nervous system (Zou *et al.* , 2013; Pereira *et al.* , 2019; Lawson *et al.* , 2019). To get a glimpse of how broadly *lin-41* may be involved in the wiring and rewiring of the nervous system, it is important to first understand what neuron types are normally expressing LIN-41 proteins at the second and third larval stages during which LIN-41 protein expression peaks in the nervous system. In this study, we use the recently developed NeuroPAL technology for nervous-system-wide neuronal identiﬁcation through whole-brain imaging (Yemini *et al.* , 2021). Worms expressing NeuroPAL display a stereotypical multicolor ﬂuorescence map for the entire hermaphrodite nervous system with unique color barcode created in each neuron, enabling identification of all neurons that also exhibit ﬂuorescence of a reporter gene in the green emission channel. Here, we determine the brain-wide expression patterns of LIN-41 proteins by engineering a reporter strain with the mNeonGreen (mNG) Cassette tagged in the endogenous *lin-41* gene using the CRISPR/Cas9 technology and co-labeling it with the NeuroPAL multicolor barcode. Although expression patterns of *lin-41* and other genes in the nervous system were recently reported by the *C. elegans* Neuronal Gene Expression Map & Network (CeNGEN) project (Hammarlund *et al.* , 2018; Taylor *et al.* , 2021), our study provides complementary and further insights into *lin-41* -expressing neurons due to two important considerations. First, CeNGEN employs bulk RNA-sequencing of individual neuron classes from L4-stage larval animals to survey molecular mapping when neuronal development and connectivity are largely complete. In contrast, our study focuses on analyzing at L2- and L3-larval stages during which *lin-41* expression peaks in the nervous system to maximize our ability to identify the *lin-41* -expressing neurons. Second, CeNGEN largely relies on cell sorting followed by RNA expression profiling, which might mask the protein expression of some genes in certain neurons where they undergo post-transcriptional gene regulation. For example, translation and stability of *lin-41* mRNAs are known to be regulated by the *let-7* microRNA. In contrast, our study reveals endogenous LIN-41 protein expression levels and their localization patterns in neurons brain-wide.

In summary, we have identified 276 LIN-41 protein-expressing neurons (Extended data, Table 1). Most of these LIN-41 protein-expressing neurons were also confirmed by a *lin-41* promoter driving GFP reporter, which is under control by a constitutive *unc-54* 3’UTR (Figure 1). This consortium of neurons represents 91% of all hermaphrodite neurons and includes 87 neurons that were not previously reported by CeNGEN using single-cell RNA-seq (Extended data, Table 1; Taylor *et al.* , 2021). For those 87 neurons that were identified as LIN-41 protein-expressing but not *lin-41* mRNA-expressing (Extended data, Table 1), a possible explanation could be that a low-level *lin-41* mRNA expression combined with a low-level *let-7* microRNA-mediated translational repression could result in a detectable level of LIN-41 protein expression. In addition, the top 10 neurons identified by CeNGEN based on the level of *lin-41* mRNA expression are not on the top 30 neuron list identified by NeuroPAL based on the frequency by which the mNG::LIN-41 fluorescence signal can be detected. The intensity of the mNG::LIN-41 fluorescence signal among different neurons is rather similar (Figure 1, C and D). Our results show that LIN-41 (TRIM71) proteins are broadly expressed in neurons, not just in the peripheral but also in the central nervous system (Figure 1; Extended data, Table 1), suggesting a widespread role of LIN-41 (TRIM71) in timing neuronal assembly, plasticity, and maintenance. (Zou *et al.* , 2013; Chiu and Chang, 2013; Ivakhnitskaia *et al.* , 2016; Ivakhnitskaia *et al.* , 2017).

## Methods

**Strains**

*C. elegans* strains were cultured using standard methods (Brenner, 1974). All strains were grown at 20°C. Standard protocol was used for the strain constructions. Strains used in this study are listed below.

**Table:** 

XN2742	*lin-41(xr76)* [mNG::LIN-41]I ; *otIs670* [NeuroPAL markers]V
XN2797	*otIs669* [NeuroPAL markers]V; *xrEx1151[Plin-41::gfp (50ng/ul)]*
XN2803	*lin-41(xr76)* I; *vyIs56[Podr-1::TagRFP]* III
XN2557	*lin-41(xr76)* I; *xrEx1010[Pmec-3::mCherry (5ng/ul)]*
XN2540	*lin-41(xr76)* I; *xrEx997[Pser-2::mCherry (5ng/ul)]*
XN2806	*evIs82b[Punc-129::gfp]* IV; *xrEx518[Plin-41::mCherry (50ng/ul)]*

**Microscopy and NeuroPAL**

Animals were mounted on 2% agarose pads and anesthetized with 7.5 mM Tetramisole. NeuroPAL images were taken in live animals using a 40x, 1.3 NA objective on a Zeiss LSM 880 confocal microscope, equipped with 7 laser lines: 405, 458, 488, 514, 561, 594, and 633 nm. Neuron types in the head and tail regions were annotated using NeuroPAL ID software. Midbody region neurons were manually annotated. The mNG::LIN-41 fluorescence intensity was analyzed by the NeuroPAL ID software. The linear change point was used as the threshold to determine LIN-41 protein-expressing neurons (Yemini *et al.* , 2021). All other images were acquired using 40x, 1.4 NA oil objective on a Zeiss Axio M2 imager equipped with Apotome. All images were acquired at L2-L3 stages.

**Generation of the mNG::LIN-41 knock-in using CRISPR-Cas9-triggered homologous recombination**

The N-terminal mNG tagged *lin-41(xr76)* [mNG::LIN-41] allele was generated by CRISPR-Cas9 mediated genome editing using the self-excising cassette strategy (Dickinson *et al.* , 2013; Dickinson *et al.* , 2015). The *lin-41* repair template homology arms and sgRNA were designed as previously described (Spike *et al.* , 2014). The following mix was injected into N _2_ animals: the repair template: mNG^SEC^3xflag^lin-41 N term (50 ng/μl), the sgRNA and Cas9-expressing construct: Peft-3::Cas9::U6p::lin-41sgRNA-N-term (50 ng/μl), co-injection markers: Prab-3::mCherry (10 ng/μl), Pmyo-2::mCherry (2.5 ng/μl), and Pmyo-3::mCherry (5 ng/μl). The correct knock-in of the mNG marker was validated by PCR and sequencing. No noticeable phenotype, judged by normal morphology, fertility, behaviors, and growth rate, was observed in the *lin-41(xr76)* [mNG::LIN-41] animals.

## References

[R1] Brenner S (1974). The genetics of Caenorhabditis elegans.. Genetics.

[R2] Chiu H, Chang C (2013). Rejuvenating nerve cells in adults.. Aging (Albany NY).

[R3] Dickinson DJ, Ward JD, Reiner DJ, Goldstein B (2013). Engineering the Caenorhabditis elegans genome using Cas9-triggered homologous recombination.. Nat Methods.

[R4] Dickinson DJ, Pani AM, Heppert JK, Higgins CD, Goldstein B (2015). Streamlined Genome Engineering with a Self-Excising Drug Selection Cassette.. Genetics.

[R5] Hammarlund M, Hobert O, Miller DM 3rd, Sestan N (2018). The CeNGEN Project: The Complete Gene Expression Map of an Entire Nervous System.. Neuron.

[R6] Ivakhnitskaia E, Hamada K, Chang C (2016). Timing mechanisms in neuronal pathfinding, synaptic reorganization, and neuronal regeneration.. Dev Growth Differ.

[R7] Ivakhnitskaia E, Lin RW, Hamada K, Chang C (2017). Timing of neuronal plasticity in development and aging.. Wiley Interdiscip Rev Dev Biol.

[R8] Lawson H, Vuong E, Miller RM, Kiontke K, Fitch DH, Portman DS (2019). The Makorin
*lep-2*
and the lncRNA
*lep-5*
regulate
*lin-28*
to schedule sexual maturation of the
*C. elegans*
nervous system.. Elife.

[R9] Pereira L, Aeschimann F, Wang C, Lawson H, Serrano-Saiz E, Portman DS, Großhans H, Hobert O (2019). Timing mechanism of sexually dimorphic nervous system differentiation.. Elife.

[R10] Spike CA, Coetzee D, Eichten C, Wang X, Hansen D, Greenstein D (2014). The TRIM-NHL protein LIN-41 and the OMA RNA-binding proteins antagonistically control the prophase-to-metaphase transition and growth of Caenorhabditis elegans oocytes.. Genetics.

[R11] Taylor SR, Santpere G, Weinreb A, Barrett A, Reilly MB, Xu C, Varol E, Oikonomou P, Glenwinkel L, McWhirter R, Poff A, Basavaraju M, Rafi I, Yemini E, Cook SJ, Abrams A, Vidal B, Cros C, Tavazoie S, Sestan N, Hammarlund M, Hobert O, Miller DM 3rd (2021). Molecular topography of an entire nervous system.. Cell.

[R12] Yemini E, Lin A, Nejatbakhsh A, Varol E, Sun R, Mena GE, Samuel ADT, Paninski L, Venkatachalam V, Hobert O (2020). NeuroPAL: A Multicolor Atlas for Whole-Brain Neuronal Identification in C. elegans.. Cell.

[R13] Zou Y, Chiu H, Zinovyeva A, Ambros V, Chuang CF, Chang C (2013). Developmental decline in neuronal regeneration by the progressive change of two intrinsic timers.. Science.

